# Endoscopic Retrograde Cholangiopancreatography and Post Endoscopy Cholecystectomies in Pediatric Population—Longitudinal, Nationwide Data from Poland

**DOI:** 10.3390/jcm14217591

**Published:** 2025-10-26

**Authors:** Karol Deptuch, Agnieszka Szlagatys-Sidorkiewicz, Beata Koń, Michał Brzeziński

**Affiliations:** 1 Department of Pediatrics, Pediatric Gastroenterology, Allergology and Nutrition, Medical University of Gdańsk, 80-210 Gdańsk, Poland; 2 National Health Fund, Department of Analysis, Quality Monitoring and Service Optimization, 02-528 Warsaw, Poland

**Keywords:** ERCP, children, cholelithiasis, choledocholithiasis, cholecystectomy

## Abstract

**Background/Objectives:** ERCP is an established method of treating cholelithiasis; however, data on its use in the pediatric population is limited. The aim of this study was to assess the prevalence of cholelithiasis among Polish children, the number of ERCP procedures performed on them, and the time between endoscopic and surgical procedures when both were necessary. **Methods:** We performed a retrospective data analysis on Polish children hospitalized due to biliary tract pathologies (ICD-10 K80–K83) in the period of 2010–2022. **Results:** In the years 2010–2022, 15,581 hospitalizations linked to the diagnosis of K80–K83 were reported. Of these, 40.71% involved patients undergoing a surgical procedure, and 4.28% involved patients undergoing ERCP (10.15% of unique patients underwent ERCP). Females accounted for 65.91% of hospitalizations, and patients in the age group of 14–17 represented 57.31% of hospitalizations. No significant yearly trends were observed in the number of hospitalizations and ERCP procedures performed. The median time between ERCP and surgical procedures was 32 days. **Conclusions:** Both the number of ERCP procedures performed in Poland and the demography of patients are consistent with data from the literature. Further research is needed to fully understand the treatment of cholelithiasis among Polish children.

## 1. Introduction

The prevalence of cholelithiasis is considerably higher in adults than in children. Over the last few decades, however, its incidence among the pediatric population has risen significantly. Such a trend is attributed to both an increased occurrence of risk factors (such as obesity) [1,2] and widespread access to sensitive diagnostic methods [3,4].

Bile stones can be found in two primary locations: in the gallbladder or the common bile duct (CBD). Choledocholithiasis accounts for approximately 10% of the cases of symptomatic gallstone disease [5]. It should be included in differential diagnoses among patients presenting with typical abdominal pain, elevated liver biochemical tests, and common bile duct distension visualized by USG, CT, MRCP, or endoscopic techniques [6,7].

Due to the lack of data on pediatric cholelithiasis and specific guidelines, most European patients under the age of 18 are treated in accordance with recommendations for the management of cholelithiasis in adults issued by the European Association for the Study of the Liver [7]. These guidelines recommend two general approaches to cholelithiasis therapy: pharmacotherapy or surgical procedures. For patients with bile stones located in the CBD, the recommended treatment is endoscopic retrograde cholangiopancreatography (ERCP). If imaging suggests bile stones in both the CBD and gallbladder, guidelines recommend performing ERCP first, followed by cholecystectomy (ideally within 72 h from the initial procedure). In 2025 new recommendations were published by the Polish Society of Gastroenterology, Hepatology and Pediatric Nutrition and the Polish Society of Pediatric Surgery. They state that ERCP is the first line of treatment for choledocholithiasis (however it also mentions laparoscopic common bile duct exploration—LCBDE—as an alternative to ERCP), and children with both cholelithiasis and choledocholithiasis should undergo ERCP followed by laparoscopic cholecystectomy performed at the earliest possible time [8]. 

Data from a retrospective analysis of cholecystectomies performed in England indicate that the number of such procedures in children under 16 years old has tripled between the years 1997 and 2012 [9]. A similar increase was observed both in Canada and the USA [1,10]. However, there is significantly less data on performing endoscopic procedures (ERCP) as well as the time between the endoscopic procedure and subsequent surgery (cholecystectomy) in the pediatric population. This gap can significantly impact treatment effectiveness and the risk of complications, as well as direct and indirect medical costs.

In order to broaden our knowledge on the management of pediatric cholelithiasis, we reviewed data from the National Health Fund (NHF)—a national service payer, covering over 98% of the population in Poland—to calculate the exact number of procedures performed in the last decade as well as analyze the epidemiology and demography of patients suffering from choledocholithiasis. The aim of this study was to determine the prevalence of cholelithiasis among Polish children and the number of endoscopic procedures performed on them, as well as the time between endoscopy and surgery when both procedures are necessary.

## 2. Materials and Methods

### 2.1. Data Acquisition

We performed a retrospective analysis of an anonymized, curated, and preliminarily analyzed dataset collected by the Polish National Health Fund. The data we received contained information about patients between the age of 0 and 18 diagnosed with biliary tract pathologies (corresponding to ICD-10 codes of K80–K83) who had ERCP or surgery performed on them in the years of 2010–2022 (see Table A1). Unique patients were differentiated by the NHF by unique identification numbers. Additionally, we gathered data concerning concomitant diseases affecting children who underwent ERCP or cholecystectomy (based on ICD10 codes assigned to patients after discharge; Table A2).

### 2.2. Statistical Analysis

Statistical analysis was conducted in the Python 3.13.2 environment using the numpy, matplotlib.pyplot, pandas, and scipy.stats libraries. The total number of hospitalizations and the number of hospitalizations with ERCP performed were tested for normality of distribution using Shapiro–Wilk test. As both distributions were not normal (with *p* value of 0.168 and 0.458, respectively), Spearman’s correlation test was performed to assess presence of yearly trends.

## 3. Results

**Figure 1:**
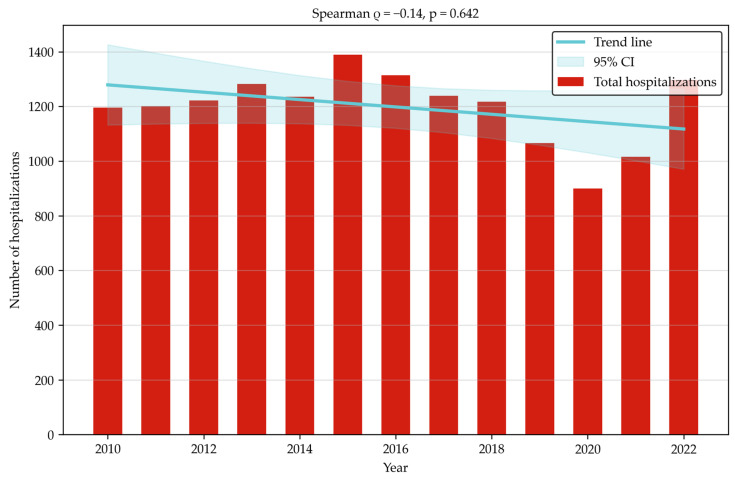
Total hospitalizations with K80–K83 diagnoses (2010–2022).

**Figure 2:**
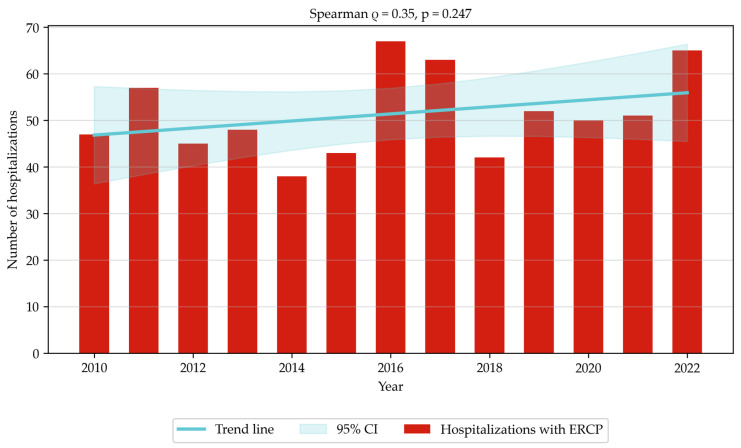
Hospitalizations with ERCP linked to K80–K83 diagnoses (2010–2022).

Between 2010 and 2022, 15,581 hospitalizations linked to a diagnosis of K80–K83 were reported (Table 1). Of these, 40.71% (i.e., 6344 hospitalizations) involved patients undergoing a surgical procedure, while 4.28% (i.e., 668) of hospitalizations involved patients undergoing ERCP. The number of unique patients treated was 6050, and 9.85% (i.e., 596) of them underwent ERCP. The highest number of hospitalizations occurred in 2015 (1390). Since then a decrease was observed, reaching the lowest value in 2020 (900; hypothetically due to restrictions in healthcare access caused by the SARS-CoV-2 pandemic). Following the pandemic, the numbers increased steadily. On average 1198.46 hospitalizations were recorded each year (SD = 132.6). (Figure 1) Similar changes are observed when analyzing hospitalizations linked to ERCP. The highest number (67) occurred in 2016, followed by a decrease to the lowest number of 42 in 2018. In subsequent years the number of ERCP-related hospitalizations increased, reaching 65 in 2022. On average 51.38 hospitalizations with ERCP performed were recorded each year (SD = 9.16). (Figure 2) Both trends were statistically insignificant.

### 3.1. Demography

According to the National Health Fund’s data, females accounted for 65.91% of hospitalizations related to biliary tract diseases among children.

**Figure 3:**
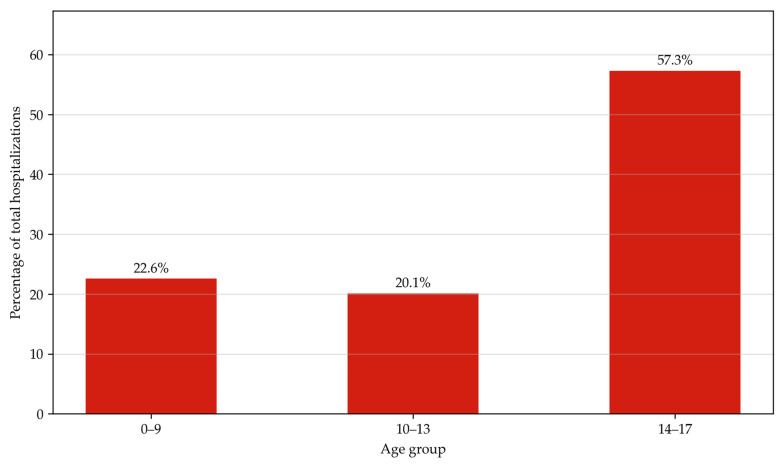
Hospitalizations with ERCP linked to K80–K83 diagnoses (2010–2022) by age group.

A total of 57.31% of hospitalizations occurred in the age group of 14–17. Children in the age group of 10–13 and 9 and below represented 20.11% and 22.58% of hospitalizations, respectively (Figure 3).

### 3.2. Concomitant Diseases

**Figure 4:**
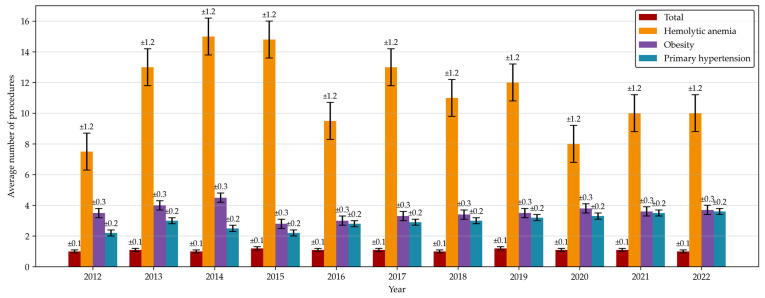
Average number of invasive procedures linked to K80–K83 diagnoses performed per patient by concomitant disease.

Between 2012 and 2022 an average of 1.12 procedures (SD = 0.02) (both ERCPs and cholecystectomies) were performed per patient. In obese patients, the ratio increased to 2.96 procedures per patient (SD = 0.26), and for patients with primary hypertension and hemolytic anemia the value increased to 3.39 (SD = 0.72) and 10.93 (SD = 2.85), respectively (Figure 4).

### 3.3. Patients Treated with Both ERCP and Cholecystectomy

Between 2010 and 2022, 271 patients underwent ERCP and cholecystectomy within a year. The gap between the two procedures depended on the patient’s diagnosis. For patients diagnosed with K80–K83, the median waiting time for cholecystectomy was 32 days (Q1 = 17 days, Q3 = 56 days).

## 4. Discussion

### 4.1. Literature Review

Cholelithiasis is a common issue among adult patients. Research indicates that up to 15% of American adults are affected by cholelithiasis during the course of their lives [11]. Nevertheless, there are no universal guidelines for the management of gallstone disease, as all the existing ones rely on data sourced from limited populations [7,12,13]. At the time of publication Polish pediatric societies have issued guidelines for treatment and diagnosis of cholelithiasis, however during the analyzed time period there were none. In contrast, in the pediatric population it is still considered a rare disease, with prevalence among children varying from 0.13% to 1.9% [14]. The relatively low number of cases results in limited data, making it a challenge to compare our survey findings with the existing literature.

By comparing the results of our research with international data, we found that the number of procedures performed in Poland is comparable to that in other countries. After adjusting for the population size, there were on average 1.36 hospitalizations with ERCP per million inhabitants per year in Poland. This compares to 1.74 hospitalizations per million inhabitants per year in the US and 0.34 in France [15,16].

The demography of patients treated in Poland aligns with worldwide trends—the American data shows that 81% of patients were female [15], whereas in a Columbian survey females made up 63.3% of the patients [17]. In contrast, French research reports males as the dominant group, as they were the subjects of 52% of ERCPs [16]. Proportions of these age groups vary from paper to paper—for instance, American research showed that over 80% of procedures were performed on patients between the age of 14 and 20 [15], while in France only 25% of patients were over the age of 15, and the median age at intervention was 10.9 years [16]. Interestingly, Czech research shows that over half of patients undergoing ERCP for biliary-related indications were under the age of 6; however, the data might be skewed by a large number of newborns being diagnosed with biliary atresia, as the researchers admit cholelithiasis is the dominant indication for ERCP among adolescents [18].

Despite the prevalence of cholelithiasis risk factors being on the rise, the statistical analysis did not shown any significant trends in the number of hospitalizations in Poland, either in total or including ERCP. Some factors that could help explain this are either that we harvested data from a relatively short period or the fact that our analysis included the years of the SARS-Cov-2 pandemic—which could have impacted the number of non-critical procedures performed. Further research is needed to explore whether the number of endoscopic procedures performed on Polish children is rising or if more and more of them are treated with other methods. That question is especially important now, since in recent years surgical approaches to bile duct exploration (the so-called “surgery-first” method) have been gaining popularity [19,20,21,22]. With this ongoing debate on whether surgery should take the place of ERCP as the first line of treatment, it is crucial to investigate how many procedures of both types are performed every year in order to assess the outcomes, complications, and cost-effectiveness of both approaches.

When choosing an endoscopy-first approach it is advised to minimize the gap between the two procedures, so that it is not longer than 72 h [7]. Adhering to this recommendation seems to be one of the biggest difficulties in the treatment of children with choledocholithiasis, as in both the Polish and French data the waiting time for cholecystectomy is much longer (respectively, 32 days (Q1 = 17, Q3 = 56) and 15 days (Q1 = 4, Q3 = 49)) [16]. Such delays could be attributed to numerous factors, such as the current operating procedures existing in hospitals, administrative difficulties, financial reasons, a reduction in symptoms after endoscopic procedures, and others. Further research is needed to investigate the issue fully and propose solutions.

It has been established that some diseases can increase the risk of cholelithiasis. Such risk factors are, for instance, hemolytic anemias or obesity [23,24,25]. Consequently, we should be able to observe either a higher percentage of patients suffering from concomitant diseases or a higher number of procedures performed per patient in these groups. And—as our data shows—the latter is the case. Our findings underscore the necessity of improving the care of patients with certain concomitant diseases in order to introduce necessary treatments early, which improves outcomes and minimizes risks of complications [7].

As a method of diagnosing and treating cholelithiasis, ERCP is both more frequently performed and associated with a higher risk of complications in patients suffering from diseases that increase the incidence of biliary obstruction compared to an otherwise healthy population. American research shows that the subjects of 13.63% of hospitalizations were obese, and such patients were at risk of having increased hospitalization times and costs [15]. French research took a different approach and did not analyze obese patients but mentioned that 8% of patients were suffering from hemolytic anemia at the time of their first ERCP [16].

### 4.2. Study Limitations

There are several limitations to our study. Firstly, as it is based on data sourced from official reports, it is subject to typical shortcomings associated with official data. For one, it is difficult to estimate how many procedures and patients were lost due to inaccurate or missing reports. This is particularly problematic when analyzing comorbidities, as some conditions (especially those seemingly less dramatic diseases such as obesity) might not be included in the patient’s history. Another issue is the fact that some ERCPs might not have been included in analysis due to the patient’s diagnosis reported to the NHF (for instance patients who had ERCP performed due to cholangitis secondary to choledocholithiasis with procedures complicated by sepsis could have been reported as diagnosed with sepsis and thus would not be included in our dataset). Furthermore, both the period analyzed and the size of the Polish sample might not be large enough for many of the otherwise occurring phenomena to be sufficiently visible (for instance it is hard to assess the influence of certain comorbidities on numbers of procedures carried out since there is too few of such patients to detect trends in the big picture). Additionally, the NHF does not share raw data on performed procedures but performs a preliminary data pooling, curation, and analysis on its own. This inhibits further analysis and renders some shortcomings in statistical analysis unavoidable. On top of that, comparative analysis between papers is impaired by variations in data collection systems and reporting methods. To combat these limitations, further research is needed, possibly using prospective questionnaires sent to endoscopic centers directly. 

## 5. Conclusions

As the data and literature review show, ERCP is an established and widely used method of treatment for biliary obstruction. It has its place in clinical guidelines and consequently should be used in accordance with them. Polish hospitals concur with these recommendations, as evidenced by the number of procedures performed, which is comparable to that in other developed countries. The demography of patients is largely consistent with the available literature; however, due to limitations in the data, the full analysis of the concomitant diseases affecting Polish patients was difficult.

In the time of an ongoing debate on shifting the treatment paradigm of cholelithiasis, data on patients’ demography and the epidemiology of choledocholithiasis is important in order to accurately assess benefits, risks, and costs associated with each treatment strategy. 

Additionally, our findings underscore the necessity for the close surveillance of patients with risk factors for cholelithiasis in order to detect the disease and its possible complications early and introduce necessary treatments.

Further research, preferably based on both prospective and retrospective data sourced from endoscopists directly, is needed to paint a full picture of the treatment of cholelithiasis among Polish children, especially in the view of newly published recommendations.

## Figures and Tables

**Table 1:** Number of hospitalizations and ERCP procedures (both in relation to K80–K83 diagnoses) in 2010–2022.

Year	Total Number of Hospitalizations	Number of Hospitalizations with ERCP Performed
2010	1196	47
2011	1202	57
2012	1222	45
2013	1283	48
2014	1236	38
2015	1390	43
2016	1315	67
2017	1239	63
2018	1218	42
2019	1066	52
2020	900	50
2021	1016	51
2022	1297	65
Total	15,581	668 (4.28%)

## Data Availability

The raw data supporting the conclusions of this article will be made available by the authors on request.

## Abbreviations

The following abbreviations are used in this manuscript:

BMI	Body mass index
CBD	Common bile duct
CT	Computed tomography
ERCP	Endoscopic retrograde cholangiopancreatography
ICD-10	International classification of diseases, 10th revision
ICD-9	International classification of diseases, 9th revision
IQR	Interquartile range
MRCP	Magnetic resonance cholangiopancreatography
NHF	National Health Fund
SARS-CoV-2	Severe acute respiratory syndrome coronavirus 2
SD	Standard deviation
US	United States
USA	United States of America
USG	Ultrasonography

## Appendix A

**Table A1:** Analyzed procedures.

ICD-9 Code	Name of the Procedure	Type of Procedure
00.94	Procedure performed using endoscopy/laparoscopy	Endoscopic/Surgery
51.10	Endoscopic retrograde cholangiopancreatography	Endoscopic
51.11	Endoscopic retrograde cholangiography	Endoscopic
51.22	Cholecystectomy	Surgery
51.239	Laparoscopic cholecystectomy	Surgery
51.85	Endoscopic sphincterotomy and papillotomy	Endoscopic
51.871	Endoscopic insertion of a biliary endoprosthesis	Endoscopic
51.88	Endoscopic removal of stone(s) from biliary tract	Endoscopic
54.21	Laparoscopy	Surgery

**Table A2:** Analyzed concomitant diseases.

ICD-10 Code	Disease
D58	Hereditary spherocytosis
E10	Type 1 diabetes mellitus
E11	Type 2 diabetes mellitus
E13	Other specified diabetes mellitus
E14	Unspecified diabetes mellitus
E66	Obesity
E78	Disorders of lipoprotein metabolism and other lipidemias
E80	Disorders of porphyrin and bilirubin metabolism
I10	Essential (primary) hypertension
